# A Digital Twin of the Coaxial Lamination Mixer for the Systematic Study of Mixing Performance and the Prediction of Precipitated Nanoparticle Properties

**DOI:** 10.3390/mi13122076

**Published:** 2022-11-25

**Authors:** Songtao Cai, Peer Erfle, Andreas Dietzel

**Affiliations:** 1 Institute of Microtechnology, Technische Universität Braunschweig, 38124 Braunschweig, Germany; 2 Center of Pharmaceutical Engineering, Technische Universität Braunschweig, 38106 Braunschweig, Germany

**Keywords:** digital twin, coaxial lamination mixer (CLM), 3D hydrodynamic flow focusing, mixing time, mixing index, nanoparticle precipitation, nanoparticle dispersity, lipid nanoparticles

## Abstract

The synthesis of nanoparticles in microchannels promises the advantages of small size, uniform shape and narrow size distribution. However, only with insights into the mixing processes can the most suitable designs and operating conditions be systematically determined. Coaxial lamination mixers (CLM) built by 2-photon polymerization can operate long-term stable nanoparticle precipitation without fouling issues. Contact of the organic phase with the microchannel walls is prevented while mixing with the aqueous phase is intensified. A coaxial nozzle allows 3D hydrodynamic focusing followed by a sequence of stretch-and-fold units. By means of a digital twin based on computational fluid dynamics (CFD) and numerical evaluation of mixing progression, the influences of operation conditions are now studied in detail. As a measure for homogenization, the mixing index (MI) was extracted as a function of microchannel position for different operating parameters such as the total flow rate and the share of solvent flow. As an exemplary result, behind a third stretch-and-fold unit, practically perfect mixing (MI>0.9) is predicted at total flow rates between 50 µL/min and 400 µL/min and up to 20% solvent flow share. Based on MI values, the mixing time, which is decisive for the size and dispersity of the nanoparticles, can be determined. Under the conditions considered, it ranges from 5 ms to 54 ms. A good correlation between the predicted mixing time and nanoparticle properties, as experimentally observed in earlier work, could be confirmed. The digital twin combining CFD with the MI methodology can in the future be used to adjust the design of a CLM or other micromixers to the desired total flow rates and flow rate ratios and to provide valuable predictions for the mixing time and even the properties of nanoparticles produced by microfluidic antisolvent precipitation.

## 1. Introduction

Proper micromixing is one of the key functions of many microfluidic chips, aiming to achieve a homogeneous mixture between different fluidic components at laminar flow conditions. The speed of mixing is of great importance for various biochemical reactions, temperature equilibration and fast biomedical sensing. Depending on the energy introduced into the system, micromixers are usually classified into two categories: passive and active micromixers [1,2,3]. Active micromixers require external influence to operate the mixing [1]. In the passive types, the mixing of the liquids is promoted by the installation of baffles, grooves, etc., which means that the mixing process does not require any external intervention [4]. Passive micromixing, which relies solely on microchannel geometry, is utilized in a large number of microfluidic systems because of high system stability. Mass transfer in passive micromixers is dominated by molecular diffusion and convection shortening the diffusion paths [5,6]. Passive micromixing can be employed to investigate a wide variety of otherwise difficult to control processes such as fast chemical and biological reactions, including protein folding/unfolding and precipitation of nanoparticles that can be utilized as a material for a wide range of applications in the electronics, chemical and pharmaceutical industries. Besides some active micromixers [7], a variety of passive microfluidic devices, including lamination and chaotic advection micromixers, droplet micromixers, hydrodynamic flow-focusing devices [8,9] and Y- and T-junctions [10,11,12,13,14] have been used for the preparation of nanoparticles [15]. Passive micromixers were utilized to produce nanoparticles in a continuous mode, in batch mode or semi-batch mode. Due to the small scales in the micromixer, fast and homogeneous micromixing at the millisecond level can be achieved, which is ideal for the preparation of nanoparticles with well-defined sizes and morphologies. The synthesis of nanoparticles in microchannels promises the advantage of small size, uniform shape and narrow size distribution, which greatly improves the quality of products. However, there are still a number of practical drawbacks and challenges to overcome in using microfluidics as a direct method for producing micro- and nanoparticles [16]. Microfluidic precipitation of nanoparticles triggered by mixing was already used to synthesize different nanoparticles [17], such as polymeric nanoparticles [18], solid lipid nanoparticles [19,20,21,22,23], drug nanoparticles [24,25,26,27,28] and liposomes [29,30,31,32]. Nanoprecipitation results from a rapid diffusion of the organic solvent and antisolvent due to the “diffusion-stranding” mechanism, which starts from the interface between the organic phase and aqueous phase [33]. The organic solution becomes supersaturated and nanoparticles precipitate. Nucleation and particle growth are key processes during nanoparticle precipitation. The nanoparticle properties will be determined by the efficiency of the mixing leading to supersaturation. Mixing efficiency can usually be controlled by the operating parameters of the total flow rate and the flow rate ratio, which in turn can be used to control the properties of the nanoparticles. While higher flow rates lead to shorter residences in the micromixer, and thereby in many cases also to a shorter mixing time, lower flow rate ratios can create thinner layers wrapped by the sheath flow in a flow focus section and thus shorter diffusion lengths. In both cases, the supersaturation level would be reached faster, and a shorter growth time for the precipitation nuclei would be given, resulting in smaller nanoparticles [16]. A practical limitation is due to the fact that most passive micromixers cannot avoid nanoparticles sticking to the microchannel walls, which leads to instabilities, particle heterogeneity and microchannel clogging during continuous operation. P. Erfle et al. recently developed the unique coaxial lamination mixer (CLM), which overcomes the problem of fouling on the walls [34]. With the technological advances in the field of two-photon-polymerization (2PP), the integration of complex 3D structures in the micromixer was possible. The CLM repeatedly stretches and folds the organic phase while keeping distance from the microchannel walls, which diminishes the fluid striation thickness and increases the interfacial area for the diffusive mass transfer [35]. This process can intensify molecular diffusion by reducing the diffusion length and increasing the contact area between different liquids to be mixed. By increasing the number of sequential 3D stretch-and-fold units, faster mixing could be achieved to a certain extent. Despite these highly advantageous properties of the CLM and the excellent and tunable properties of produced nanoparticles, a systematic investigation of the influence of all geometric parameters and all operational conditions, which will allow the optimization with respect to throughput and the of quality of specific nanoparticles, was not yet possible. To investigate this experimentally, the number of tests would be far too high. In our previous work [34], only the streamlines (only representing convection but not diffusion) were calculated. The ambition of this paper is to consider both convection and diffusion. For this purpose, the CLM with complex 3D stretch-and-fold units was turned into a digital twin allowing us to numerically investigate and to predict the mixing performance. The goal was to develop a digital tool to predict mixing times and achievable nanoparticle sizes.

## 2. Materials and Methods

### 2.1. Digital Geometry of the CLM

**Figure 1:**
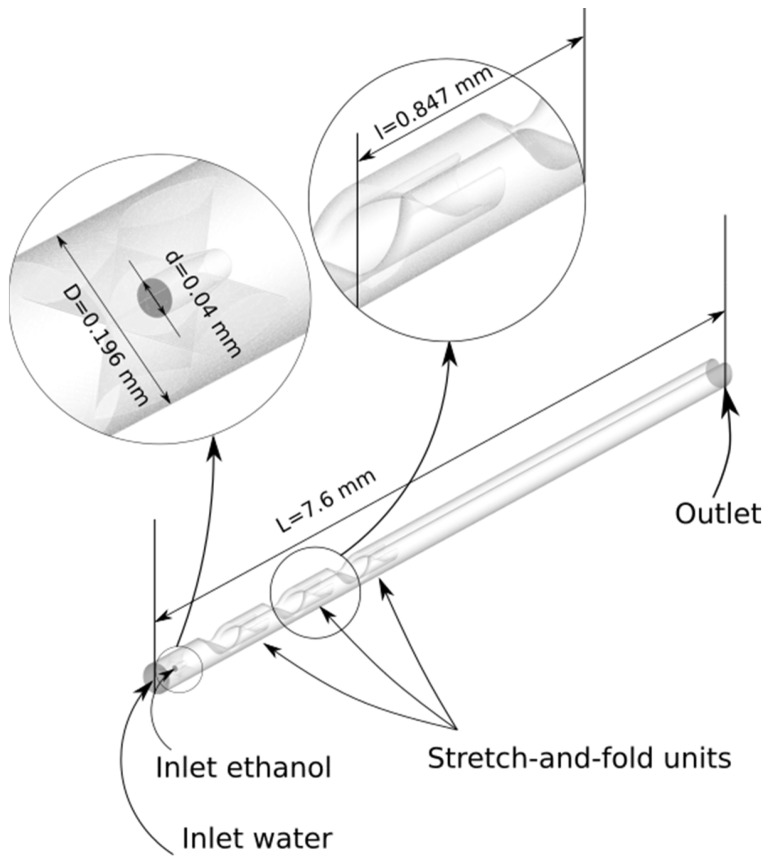
Ilustration of the 3D-CLM design with three stretch-and-fold units as flow domain for the simulations.

The considered CLM designs were created using commercial 3D modeling software SolidWorks (SolidWorks 2020 Education Edition, Dassault Systèmes SolidWorks Corp., Waltham, MA, USA). An example is given in Figure 1, which shows the geometry of the flow domain in accordance with the earlier experimental work (three identical 3D stretch-and-fold units and microchannel length of 7.6 mm), the diameters of the water and ethanol inlets were 0.196 mm and 0.04 mm, respectively [34]. The length of single stretch-and-fold units was 0.847 mm. All geometrical features can be easily adjusted to consider other design modifications.

### 2.2. Flow and Mixing Analysis

Liquids in microchannels can be considered as continuous fluidic media. The energy equation is not to be considered because of the chemical inertness of the system and the negligibility of thermal effects of dissolution. The flows are assumed to be laminar. The working fluids are considered to be incompressible and Newtonian, in steady-state and not affected by gravity. The fluid field simulations are based on the continuity Equation and the Navier–Stokes Equation [36]:

(1) $$\nabla \cdot \vec{u}=0,$$

(2) $$\rho \vec{u}\cdot \nabla \vec{u}=-\nabla p+\mu \nabla^{2}\vec{u},$$

where the symbols $\vec{u}$, ρ, μ and p denote the velocity vector, the density and the dynamic viscosity of the fluid and the pressure, respectively. It is assumed that the influence of gravity and other body forces can be neglected. The concentration fields can be calculated based on the convection-diffusion equation [37]:

(3) $$\vec{u}\cdot \nabla c=D\nabla^{2}c,$$

where c and D denote solution concentration and the diffusion coefficient, respectively. This Equation (3) can be solved together with Equations (1) and (2) in order to achieve coupling between the velocity field and the concentration field. Ethanol and water at 293.15 K are considered as the solvent and antisolvent fluids with the physical properties given in Table 1.

Assuming that no chemical reaction takes place, the viscosity of water-ethanol mixtures is given as the mass-weighted sum of the viscosities of the two components. The complex geometry of the mixer results in a high computational cost, so the simulation is simplified by assuming that minimal volume changes due to the dissolution of ethanol in water and any influences of the dissolved or precipitated lipid on the flows can be neglected. A non-linear viscosity model has been proposed in the literature [38,39], but only in connection with a simple geometry. Our investigations are focused on the role of complex geometry on mixing, so the estimates of mixing are too weakly affected by these simplifications to exert a non-negligible influence on the results. 

Uniform velocities at the inlets, zero pressure at the outlet and slip-free boundary conditions at the microchannel walls are assumed. 100% water is assigned to the antisolvent inlet and 100% ethanol to the solvent inlet. 

The microchannel with complex 3D geometry for stretch-and-fold units was discretized by 3D tetrahedral elements. The numerical analyses of fluid flow and mixing were performed using commercial CFD software (ANSYS FLUENT 2020 R1 from ANSYS, Inc., Canonsburg, PA, USA) operated in double precision mode. The SIMPLEC (Semi implicit pressure linked equation-consistent) algorithm was used as the solution method to couple the pressure and velocity fields [40]. The Green–Gauss node-based method was used for gradient discretization. The spatial discretization PRESTO! (PREssure STaggering Option) scheme was employed to solve the spatial pressure discretization [41]. The second-order upwind schemes discretization was employed for calculating the momentum equation to achieve a more accurate solution. To reduce numerical diffusion, the higher-order approximation schemes for the discretization of advection terms such as Third-Order MUSCL (Monotonic Upstream-centered Scheme for Conservation Laws) scheme were applied in addition to fine meshing [42]. Iterations were continued until the relative residuals for the convection-diffusion equation, the pressure equation and the momentum equation fell below a threshold of 10^−4^.

### 2.3. Quantification of Progressive Mixing

To evaluate the mixing performance, a quantitative measure needs to be defined. From the various statistical models found in the literature, the mixing index (MI) defined in Equation (4) [43] was selected for this study to quantitatively describe the progression of mixing:

(4) $$\mathrm{MI}=1-\sqrt{\frac{\sigma^{2}}{\sigma_{max}{}^{2}}}\;,$$

where MI varies from 0 (σ = $\sigma_{max}$, unmixed) to 1 (σ = 0, completely mixed). The solution concentration variance σ was calculated on selected cross sections with planes normal to the flow direction according to Equation (5) [43]:

(5) $$\sigma =\sqrt{\frac{1}{n}\overset{n}{\underset{i=1}{\sum }}{\left(c_{i^{-}}\bar{c}\right)}^{2}},$$

where n, $\bar{c}$ and $c_{i}$ are the number of sampling points in the cross sections, concentration averaged over the cross-section and the actual mass fraction at sampling point i, respectively. $\sigma_{\max}$ denotes the maximum concentration variance over the entire data range mostly at the inlet (typically at the point of first contact between the two working fluids) [44]. A MATLAB (MATLAB Release Version R2019b, MathWorks, Inc., Natick, MA, USA) code was written, which automatically evaluates the concentration field obtained in the simulation and calculates MI. With 0.9<MI<1, the mixing can be considered complete [45].

As was concluded from the earlier CLM experiments, the nanoparticle properties are determined by the mixing time [34]. The elapsed residence time $t\left(x\right)$ at the position x could be estimated using the following equation:

(6) $$t\left(x\right)=\frac{V\left(x\right)}{Q},$$

where $V\left(x\right)$ is the microchannel volume between the position x and the position of the injection nozzle outlet $x_{\mathrm{cont}}$, which allows the first contact between the two liquids. Q is the total flow rate in the microchannel. The elapsed residence time at the earliest position with complete mixing $x_{\min}$, is considered as the mixing time $t_{\mathrm{mix}}$.

## 3. Results and Discussion

### 3.1. Mesh Convergence Testing

**Figure 2:**
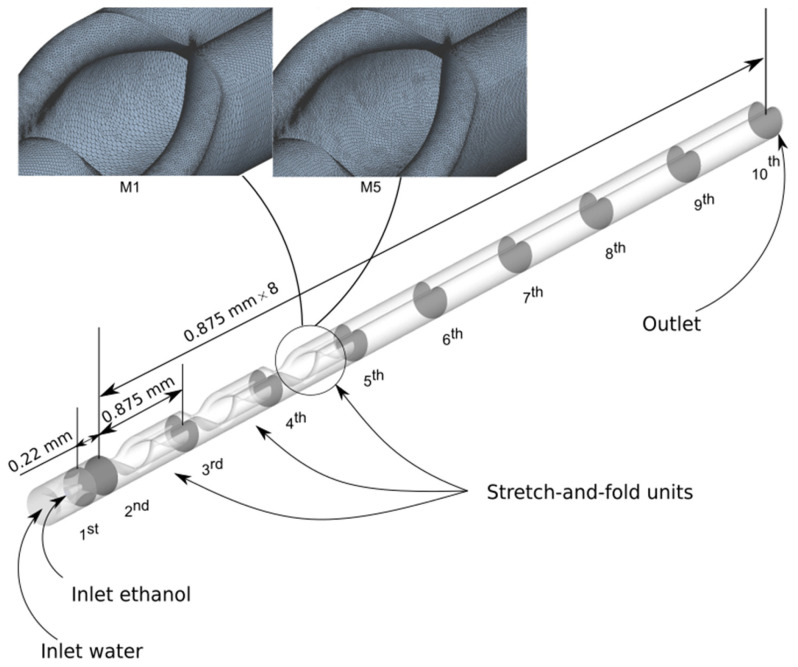
Illustration of the positioning of ten cross-sectional planes in which MI values were obtained. From the second plane onwards, the positioning is equidistant. Zoomed views of a coarser (M1) and a finer mesh (M5) at astretch and fold unit are shown.

**Figure 3:**
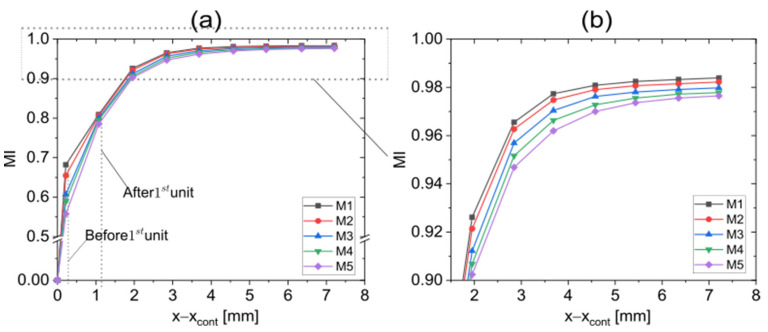
(**a**) Mesh convergence testing for a CLM having three stretch-and-fold units operated with a total flow rate of 36 µL/min and a flow rate ratio of 10% to determine the required mesh level. MI values are plotted as function of the position in the microchannel for different mesh levels. (**b**) Zoom in of an MI range between 0.9 and 1.

The complex 3D geometries within the microchannel increase the challenge of generating a mesh with sufficient quality. The 3D unstructured tetrahedral mesh with inflation layers was used. To reduce the numerical diffusion to an acceptable level, ensuring sufficiently accurate simulation results, in each computational domain, the mesh was gradually refined. This made the important results almost impervious to progressive refinements, and given our limited computer resources, any further refinement meant only superfluous effort. This grid verification was carried out at a fixed total flow rate of 36 µL/min and a flow rate ratio of 10% with a CLM having three stretch-and-fold units as illustrated in Figure 2. A sequence of planes in which the local MI can be determined was introduced. The first plane was located at $x_{\mathrm{cont}}$, the injection nozzle outlet. Further planes were placed at equidistant positions (distance = 0.875 µm) so that stretch-and-fold units fall midway between them. MI values obtained for different meshing levels are shown in Figure 3 as a function of distance to the first plane. Table 2 lists how the grid mean size ∆x reduces from meshing levels M1 to M5. For all levels, MI increases along the microchannel, but for coarse meshes, MI values appear slightly higher than for the finer meshes, indicating remaining numerical diffusion. MI initially varies strongly with the mesh density, but as the fluid passes more and more mixing units, better convergence is observed. Behind the first unit, the obtained MI values become sufficiently accurate, and the position after which the MI reaches 0.9 practically becomes insensitive to further refinements. At higher flow rates, the cell Péclet number, defined as ${\mathrm{Pe}}_{\mathrm{cell},\mathrm{ave}}=V_{\mathrm{ave}}*∆x/D$ ($V_{\mathrm{ave}}$ is the average velocity in the flow domain), increases, indicating that convective mixing more and more dominates. As a consequence, in addition the contribution of numerical diffusion to the MIs behind the first mixing unit can be neglected. With ${\mathrm{Pe}}_{\mathrm{cell},\max}$ < 2 numerical diffusion is completely eliminated, but good agreement between the numerical solution and experimental results was even obtained for ${\mathrm{Pe}}_{\mathrm{cell},\max}$ = 4000 [46]. In addition, next to ${\mathrm{Pe}}_{\mathrm{cell},\mathrm{ave}}$, the maximum cell Péclet numbers ${\mathrm{Pe}}_{\mathrm{cell},\max}$ are listed in Table 2 based on the maximum velocity $V_{\max}$ = 0.154471 m/s in the mixing channel. Mesh level M5 (${\mathrm{Pe}}_{\mathrm{cell},\max}$ = 20) was in the following simulations. Since a high-order discretization scheme (Third-Order MUSCL) was chosen, the remaining influences of numerical diffusion were further reduced without an extensive increase in computer power. 

### 3.2. Effect of Multiple Stretch-and-Fold Units on the Mixing Performance

**Figure 4:**
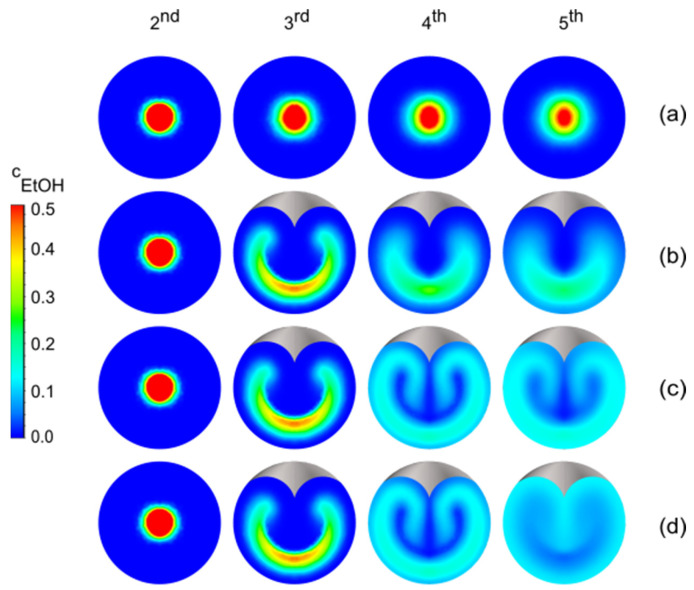
Concentration distributions of ethanol $c_{\mathrm{EtOH}}$ on cross-section areas from the second plane to the fifth plane as obtained with digital CLMs holding a varied number of stretch-and-fold units. It has to be mentioned that the concentrations of ethanol resulting from convection and diffusion are shown, while in earlier work [34], only the streamlines (representing only convection but not diffusion) were given. (**a**) without stretch-and-fold units (**b**) one stretch-and-fold unit between the second plane and the third plane (**c**) an additional second stretch-and-fold unit between the third plane and the fourth plane (**d**) an additional third stretch-and-fold unit between the fourth plane and the fifth plane. The concentrations are color coded as the dimensionless ratio of the mass of ethanol to the total mass of the mixture.

Figure 4 shows the organic phase concentrations in four planes as obtained for digital CLMs with up to three stretch-and-fold units. At the second plane, the two fluids are almost unmixed. The first stretch-and-fold unit forms a thin, curved fluid shell whose shape is blurred by diffusive mixing, indicating that one unit already improves the mixing performance considerably. With adding stretch-and-fold units leading to multiple laminations, the mixing is further accelerated.

**Figure 5:**
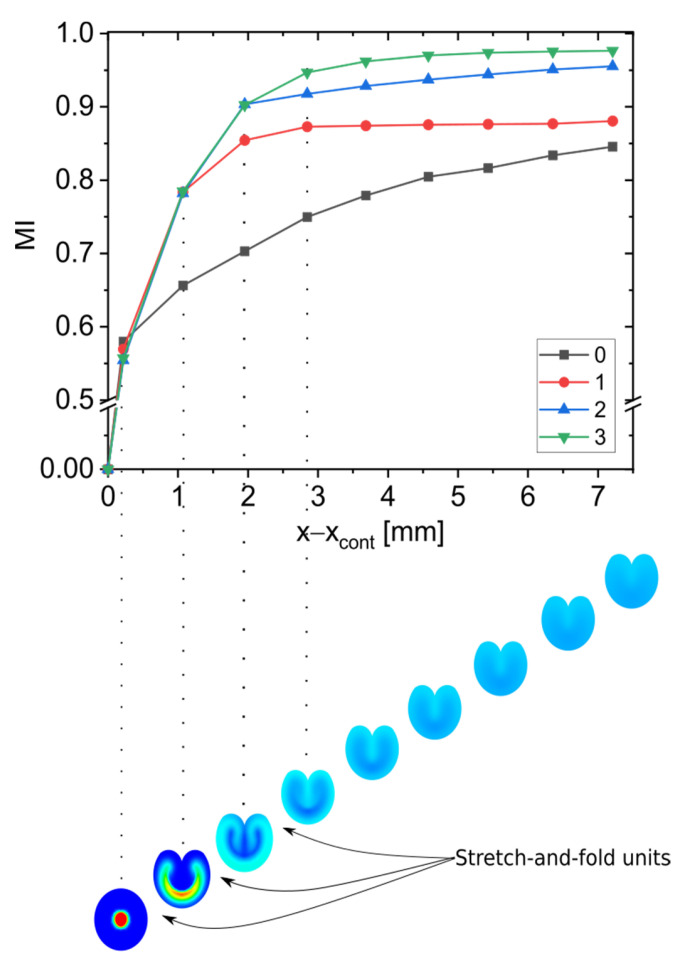
MI versus the positions in flow direction at a total flow rate of 36 µL/min with a 10% flow rate ratio for a varied number of stretch-and-fold units. The color of data points indicates the number of stretch-and-fold units. The investigated planes with exemplary concentration fields are indicated below the diagram to indicate their position together with the positions of the stretch-and-fold units. As in Figure 4 the concentrations of ethanol resulting from convection and diffusion are shown, while in earlier work [34], only the streamlines (representing only convection but not diffusion) were given.

As shown in Figure 5, with adding stretch-and-fold units, a more intense mixing is observed behind the additional unit. Already, in a CLM with two stretch-and-fold units, an almost perfect mixing (MI>0.9) is reached.

### 3.3. Effect of the Flow Rate Ratio on the Mixing

**Figure 6:**
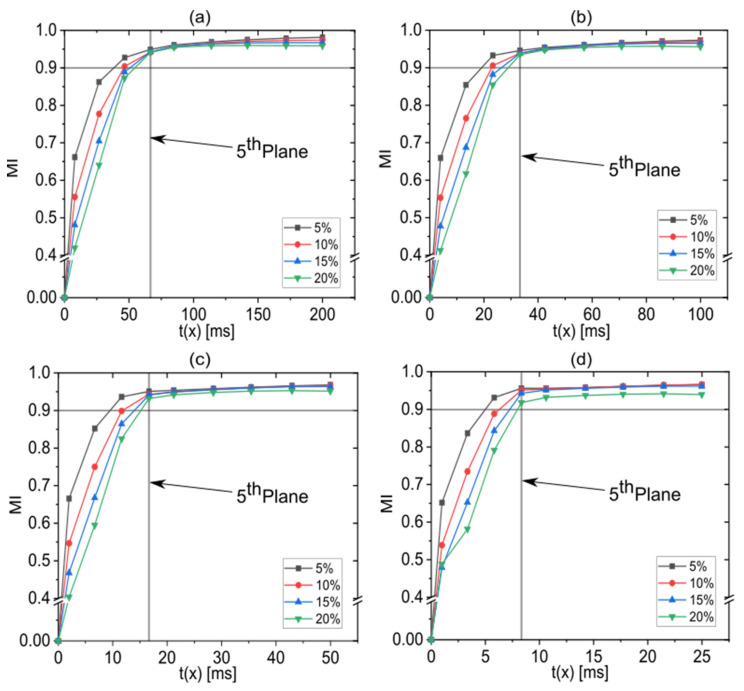
MI as function of elapsed residence time for flow rate ratios varied between 5% and 20% at constant total flow rates of (**a**) 50 µL/min, (**b**) 100 µL/min, (**c**) 200 µL/min and (**d**) 400 µL/min. Straight vertical lines indicate the position of the fifth plane at which the third stretch-and-fold unit is passed and after which the curves practically overlap. For data above the straight horizontal lines practically complete mixing (MI > 0.9) is achieved.

**Figure 7:**
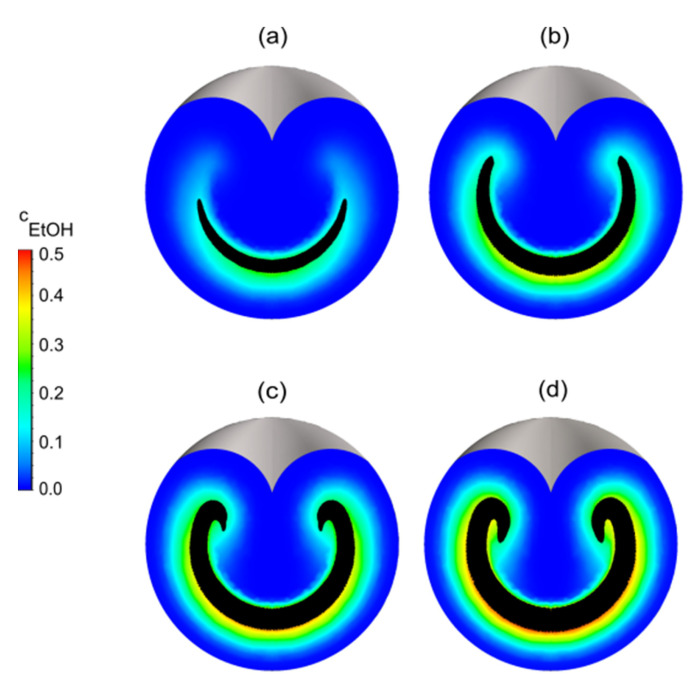
Concentration distributions (color coded) of ethanol $c_{\mathrm{EtOH}}$ at the third plane placed behind the first stretch-and-fold unit for varied flow rate ratios: (**a**) 5%, (**b**) 10%, (**c**) 15% and (**d**) 20%. As in previous figures, the concentrations of ethanol resulting from convection and diffusion are color coded. The black colored areas, however, indicate the area occupied by the solvent when neglecting diffusion as obtained by streamline simulation. Note that at the lower flow ratios, too few streamlines are generated in the upper half of the cross sections to become recognizable as very thin black areas.

A digital CLM with three stretch-and-fold units was used to investigate the influence of the flow rate ratio. Figure 6 shows MI versus elapsed residence time for varied flow rate ratios while total flow rates were kept constant. The MI values are lower at higher flow rate ratios, but are practically indifferent as soon as the fifth plane is reached, in other words, after the third stretch-and-fold unit is passed. At higher total flow rates, MI increases faster because the stretch-and-fold units are reached earlier. As shown in Figure 6d, the MI value at the first plane at a high flow rate of 400 µL/min with a flow rate ratio of 20% is a little bit higher than the MI value at the first plane at a high flow rate of 400 µL/min with a flow rate ratio of 15%. It can be assumed that this is a consequence of numerical diffusion [47,48] appearing at such high flow rates. This could be suppressed with an extremely-fine mesh [49,50,51,52,53]. At low flow rate ratios, MI increases faster, as can be explained looking at the concentration distributions of ethanol on cross-sections placed after passing the first stretch-and-fold unit (Figure 7). The thickness of the solvent phase layer is controlled by the flow rate ratio and influences the time required for interdiffusion according to $t=l^{2}/2d$ [54]. For the high flow rate of 400 µL/min, the MI value remains slightly lower for a 20% flow rate ratio even after the third unit is passed, but for the total flow rates from 100 µL/min to 200 µL/min, the MI values reach the same level for all considered flow rate ratios. The fastest mixing was observed at a total flow rate of 400 µL/min at a 5% flow rate ratio. In this case, mixing was practically completed already before the fifth plane was reached, while in all other cases, mixing was not completed before the fifth plane (after the third stretch-and-fold unit) was passed. $t_{\mathrm{mix}}$ increased to 54 ms for the lowest considered total flow rate of 50 µL/min combined with the highest considered flow rate ratio of 20%. The total residence times (required to pass through the entire microchannel) were 200 ms, 100 ms, 50 ms and 25 ms, at the total flow rates of 50 µL/min,100 µL/min, 200 µL/min and 400 µL/min, respectively. 

### 3.4. Effect of the Total Flow Rate on the Mixing Performance

**Figure 8:**
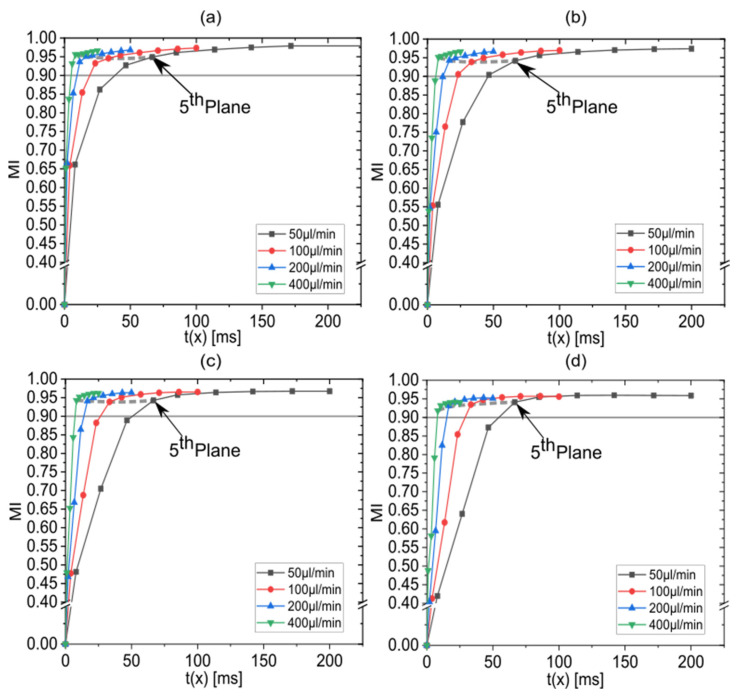
MI versus elapsed residence time for varied total flow rates of 50 µL/min, 100 µL/min, 200 µL/min and 400 µL/min at flow rate ratios of (**a**) 5%, (**b**) 10%, (**c**) 15% and (**d**) 20%. Above the straight gray horizontal lines, practically complete mixing is achieved. Data points corresponding to the fifth plane are connected with dotted gray lines.

In order to illustrate how total flow rates influence MI, the data already presented in Figure 6 are now grouped for constant flow rate ratios as shown in Figure 8. With higher total flow rates, the stretch-and-fold units are reached at an earlier point in time. All curves show saturation towards a value of MI≈0.97. In general, for a CLM with three stretch-and-fold units, $t_{\mathrm{mix}}$ is below the total residence time for all considered flow rates, and a high throughput can be realized without compromising mixing performance. Since saturation is already reached after the third stretch-and-fold unit, adding another unit would not further reduce the mixing time.

**Figure 9:**
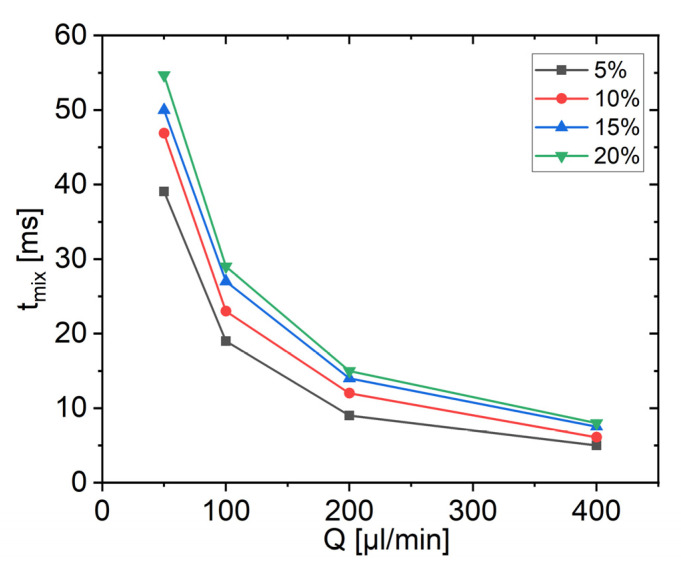
$t_{\mathrm{mix}}$ obtained for the digital CLM with three stretch-and-fold units in dependence on the total flow rate Q for varied flow rate ratios.

The mixing time is considered to be a key physical parameter for controlling nanoparticle size and size distribution (typically characterized by polydispersity index PDI) in nanoprecipitation. The mixing time is therefore suitable for comparing the performance of mixing processes and mixing devices. The development of micromixers with ever shorter mixing times is crucial for the production of monodisperse nanoparticles. It is to be expected that not only the value of the total residence time, but also the mixing time decreases strongly when the value of the total flow rate Q increases. Figure 9 shows $t_{\mathrm{mix}}$, which results from the simulations, as described in Figure 8 in the previous section, as a function of the total flow rate Q for different flow rate ratios with three stretch-and-fold units. An interpolation of data with the B-spline method was applied. It shows values of $t_{\mathrm{mix}}$ in the range of 5~54 ms, depending on the flow rate ratio. $t_{\mathrm{mix}}$ reduces with higher total flow rates but also with lower flow rate ratios. It was observed that, at a total flow rate of 400 µL/min and a flow rate ratio of 5%, a $t_{\mathrm{mix}}$ of as low as 5 ms is reached. Since $t_{\mathrm{mix}}$ can be easily controlled by the total flow rate and the flow rate ratio, the next important question is to what extent it can also be used to control nanoparticle properties during microfluidic precipitation.

### 3.5. Correlation between Nanoparticle Properties and the Mixing Time

**Figure 10:**
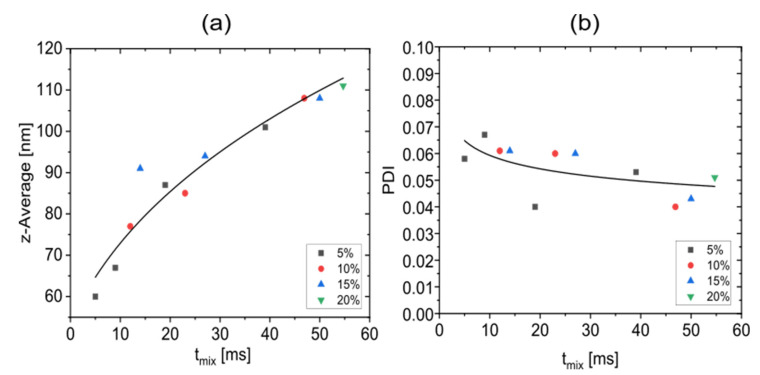
The correlation between the mixing times obtained with the digital CLM twin with three stretch-and-fold units and experimental data (each data point represents the average size of three measurements) obtained with the real CLM [28]. The data points include all considered flow rate ratios. (**a**) Correlation with nanoparticle sizes (z-average). The drawn curve shows a fit to the data points assuming $z_{\mathrm{average}}{}^{3}=z_{0}{}^{3}+{k*t}_{\mathrm{mix}}$. (**b**) The correlation of mixing time with the mean (from three experiments obtained) PDI values which are more scattered, is much weaker. The drawn curve therefore indicates a trend only.

To investigate the predictive power of $t_{\mathrm{mix}}$ for nanoparticle precipitation result, previous experimental results of nanoparticle precipitation in the CLM [34] were used, while total flow rates agree with those of the digital twin. Experiments leading to fouling or blocking were not considered. Figure 10a shows the correlation between the average size of experimentally obtained lipid nanoparticles with $t_{\mathrm{mix}}$ for different flow rate ratios. It shows that the mean size of lipid nanoparticles strongly correlates with $t_{\mathrm{mix}}$ regardless of the flow conditions. Nanoparticle sizes below 100 nm are obtained for $t_{\mathrm{mix}}\leq 40\mathrm{ms}$. The smallest experimentally found nanoparticle size of 60 nm corresponds to $t_{\mathrm{mix}}=5\;\mathrm{ms}$. It is known from the literature that the dependence of nanoparticle size from $t_{\mathrm{mix}}$ can be described as $z_{\mathrm{average}}{}^{n}=z_{0}{}^{n}+{k*t}_{\mathrm{mix}}$, where $z_{\mathrm{average}}$ is the mean particle size at time t, n the growth exponent, $z_{0}$ the size at the onset of particle growth and k the nanoparticle growth rate [55]. This is also consistent with $\frac{{\mathrm{dz}}_{\mathrm{average}}}{\mathrm{dt}}\sim z_{\mathrm{average}}{}^{1-n}$, the nanoparticle growth law from Burke [56]. For *n* = 3, the best fit to our data is observed for $z_{0}=53.46\;\mathrm{nm}$ and $k=3.9*10^{-1}{\mathrm{nm}}^{3}/\min.$ Figure 10b shows the correlation of experimentally obtained mean PDI values [34] with $t_{\mathrm{mix}}$. The PDI values range from 0.04 to 0.067, which in all cases indicate desirable monodispersity (defined as PDI<0.07 [34]). However, a correlation with $t_{\mathrm{mix}}$ is not as clear and strong as for nanoparticle sizes. Nevertheless, it is apparent as a trend that lower PDI values are more likely at lower flow ratios and slower mixing. While lower flow rate ratios lead to smaller nanoparticle sizes and also smaller PDI values, higher total flow rates, which also reduce the mixing times, seem to lead to smaller nanoparticles but additionally, to slightly higher PDI values. 

## 4. Conclusions and Outlook

A digital twin of a recently developed CLM was established to investigate the mixing performance in dependence on operational conditions. A three-dimensional CFD model has been developed, which can reveal detailed information about the flow fields and the concentration fields at each position within the CLM depending on operating parameters, the total flow rate and the flow rate ratio. In a next step, an algorithm was established for calculating the mixing index representing the degree of mixing at multiple cross sections of the CLM. Finally, the mixing index values obtained at certain microchannel positions were considered as a function of time to determine $t_{\mathrm{mix}}$, at which complete mixing is reached. It showed that a CLM with three stretch-and-fold units can realize complete mixing at all considered operational conditions, but $t_{\mathrm{mix}}$ can be reduced by increasing the total flow rate, which makes the CLM an ideal device for high throughput. Further, $t_{\mathrm{mix}}$ also reduces with decreasing the flow rate ratio. An important result of the digital twin with three stretch-and-fold units is also that, no further reduction of $t_{\mathrm{mix}}$ can be achieved by adding another unit. The mixing time obtained with the digital CLM was found to strongly correlate with nanoparticle sizes previously obtained in experiments with the “real” CLM in the form of a proportionality to the thirdroot of $t_{\mathrm{mix}}$. However, the nanoparticle size distributions obtained in the experiments in the form of PDI values do not strongly correlate with $t_{\mathrm{mix}}$. A reduction of $t_{\mathrm{mix}}$ by decreasing the flow rate ratio does lead to narrower size distributions, as the digital twin would lead us to expect. However, increasing the total flow rate, which also reduces $t_{\mathrm{mix}}$ in the digital twin, tends to slightly broaden the nanoparticle size distribution in the experiments. It still needs to be investigated in more detail which processes are responsible for this. The correlation with earlier experiments could nevertheless confirm that, $t_{\mathrm{mix}}$ is the key parameter determining properties of nanoparticles produced by solvent antisolvent nanoprecipitation. Furthermore, the systematics of the digital micromixer twin has proven to be very useful. ln principle, it can be applied to almost all forms of micromixers, in particular, also the recently developed horseshoe lamination mixer [57]. It can also help to better understand the mixing processes, to rely less on trial and error during the development, and to make early predictions of micromixer performance before they are realized by microfabrication methods. 

## Figures and Tables

**Table 1:** Physical properties of the two considered working fluids at 293.15 K [32].

Fluid	Density [kg m^−3^]	Viscosity [kg m^−1^s^−1^]	Diffusivity [m^−2^s^−1^]
Water	9.998 × 10^2^	0.9 × 10^−3^	1.2 × 10^−9^
Ethanol	7.890 × 10^2^	1.2 × 10^−3^	1.2 × 10^−9^

**Table 2:** Influence of meshing on ${\mathrm{Pe}}_{\mathrm{cell},\mathrm{ave}}$ and ${\mathrm{Pe}}_{\mathrm{cell},\max}$.

Meshing Level	∆x (µm)	${\mathrm{Pe}}_{\mathrm{cell},\mathrm{ave}}\left(\sim V_{\mathrm{ave}}\right)$	${\mathrm{Pe}}_{\mathrm{cell},\mathrm{ave}}\left(\sim V_{\max}\right)$
M1	5	46	644
M2	4.2	38	541
M3	3.4	31	438
M4	2.6	23	335
M5	2.2	20	283

## Data Availability

The data that support the findings of this study are available from the corresponding author upon reasonable request.
